# Supplementary material for: Novel EDGE encoding method enhances ability to identify genetic interactions
Source: PLoS Genet. 2021 Jun 4;17(6):e1009534. doi: 10.1371/journal.pgen.1009534 (PMC8208534; doi:10.1371/journal.pgen.1009534)
Supplement: S1 Text — (DOCX) [file pgen.1009534.s001.docx]

*Biallelic Model Simulator (BAMS)*

BAMS is a simulator tool to simulate datasets with two SNPs exhibiting main effects and/or interaction effects. This tool was used to compare a novel EDGE encoding (Hall et al., accepted *PLoS Genetics*) to traditional additive, dominant, recessive, and codominant genetic encodings and allows simulation of SNPs with diverse underlying genetic models (e.g., additive, dominant, recessive) with a wide range of parameters including sample size, case/control ratio, minor allele frequency (MAF), and penetrance. The simulation script generates two independent, biallelic SNPs in Hardy-Weinberg equilibrium according to given minor allele frequencies. With the genotype generated, a phenotype is determined using a traditional 9-cell penetrance table. The simulation script can generate both quantitative and dichotomous phenotypes; in the case of quantitative phenotypes, the calculated dependent variable is a random sample from a normal distribution with variance 1 and a mean given by the penetrance table. In the case of dichotomous phenotypes, each cell in the penetrance table represents the probability of being affected, and rejection sampling is used to generate the requested number of cases and controls.

The penetrance table used in the simulation script may either be provided directly, or it can be generated according to the model:
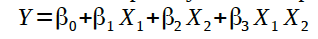
In the previous formula, *Χ_i_* represents the given normalized effect for each genotype for SNP i. As an example, “0,0,1” represents a recessive effect, while “0,0.5,1” represents an additive effect.

In order to normalize the amount of detectable effect in the interaction term across different models, the simulation script also offers an option to automatically scale the penetrance table according to the amount of detectable signal of the interaction term using a codominant encoding. To do this, we construct a 9-row regression with each row corresponding to a cell in the penetrance table. The model matrix will be defined to be the intercept term along with the codominant encoding (note: Any linearly independent encoding will work for our purposes; we chose codominant for ease of understanding) of both SNPs, yielding 5 columns. The dependent variable is the normalized value of the penetrance table. Normalization in this case does not change the result, but it does allow for the same starting point when applying the adjustment factor. Any dependent variable that maintains collinearity following transformation, an affine transformation (multiply by a constant, then add a constant), of the given penetrance values will yield identical variance explained.


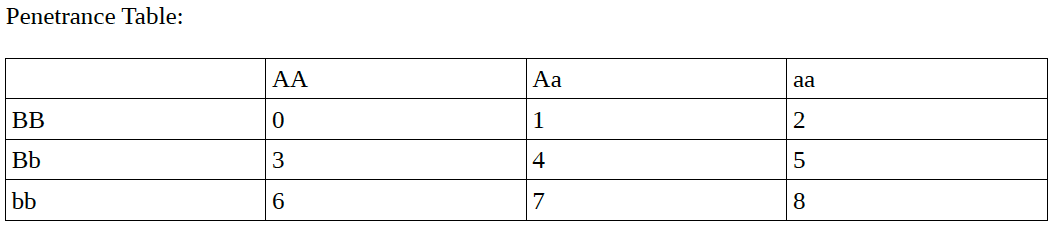


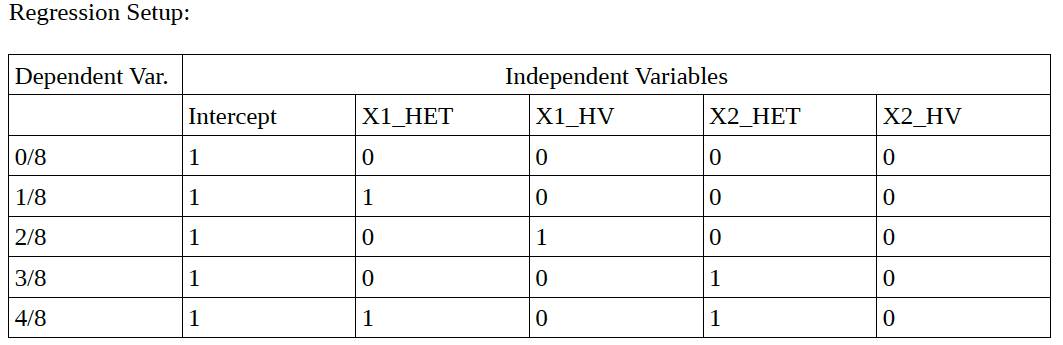

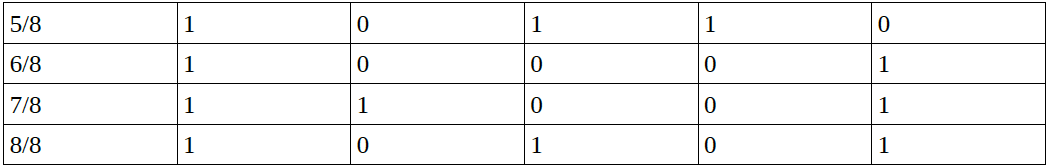


Because there are only have nine rows above, we can see that including the interaction terms between the two SNPs will cause the regression to fit the data, as there will be nine columns of independent variables (1 intercept, 2 each of 2 columns of main effects and 4 columns of interaction effects). Thus, any unexplained variance in the above model is due entirely to the missing interaction terms.

To account for the impact that minor allele frequency can have on the amount of signal, we perform a weighted linear regression on the above data, where the weights are defined by the probability of an individual having a given genotype under the assumption of two independent SNPs in Hardy-Weinberg Equillibrium in a population of infinite size. The amount of “signal” available to find is determined by the weighted residual standard error. In the case of a perfectly predictive regression, as would be the case in a model with only main effects, this process is repeated with the model matrix consisting of only the intercept term. The weighted residual standard error is defined as:


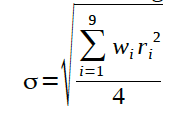


Where *r_i_* is are the residuals and the *w_i_* are the weights, as defined previously.

In the case of simulating a quantitative phenotype, the new penetrance table is defined to be the normalized penetrance table times
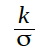
for some configurable value k, defined to be the “signal to noise ratio.”

In the case of simulating a dichotomous phenotype, we want the probabilities calculated such that the odds ratio between the least penetrant cell and the most penetrant cell is our expression
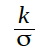
as above. Additionally, we want the least penetrant cell and the most penetrant cell to be equally close to 0 and 1, respectively, with all of the probabilities scaling linearly. We define the minimum probability as:


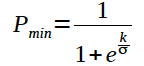
, and then the new penetrance table is now defined as:


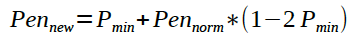


To prevent model non-convergence of regression using the simulated datasets, we allow the user to choose a baseline risk (i.e., the simulated samples with the lowest risk genotypes are provided a user-determined probability of being labeled “case”) and maximum risk (i.e., the maximum probability of being labeled “case” for simulated samples with highest-risk genotype). Therefore, penetrance can be tested by varying the baseline to maximum risk ratios.
