# Supplementary figures and images for: Novel EDGE encoding method enhances ability to identify genetic interactions
Source: PLoS Genet. 2021 Jun 4;17(6):e1009534. doi: 10.1371/journal.pgen.1009534 (PMC8208534; doi:10.1371/journal.pgen.1009534)

**S1 Figure.** Impact of MAF on alpha value.

**
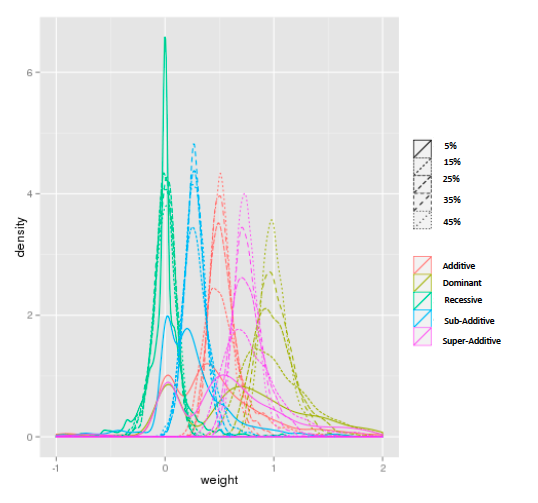
**

Supplement: S1 Fig — (DOCX) [file pgen.1009534.s002.docx]
