# Supplementary material for: Novel EDGE encoding method enhances ability to identify genetic interactions
Source: PLoS Genet. 2021 Jun 4;17(6):e1009534. doi: 10.1371/journal.pgen.1009534 (PMC8208534; doi:10.1371/journal.pgen.1009534)

**S2 Figure.** Results of the multi-encoding GWAS for age-related cataract, glaucoma, and resistance hypertension.


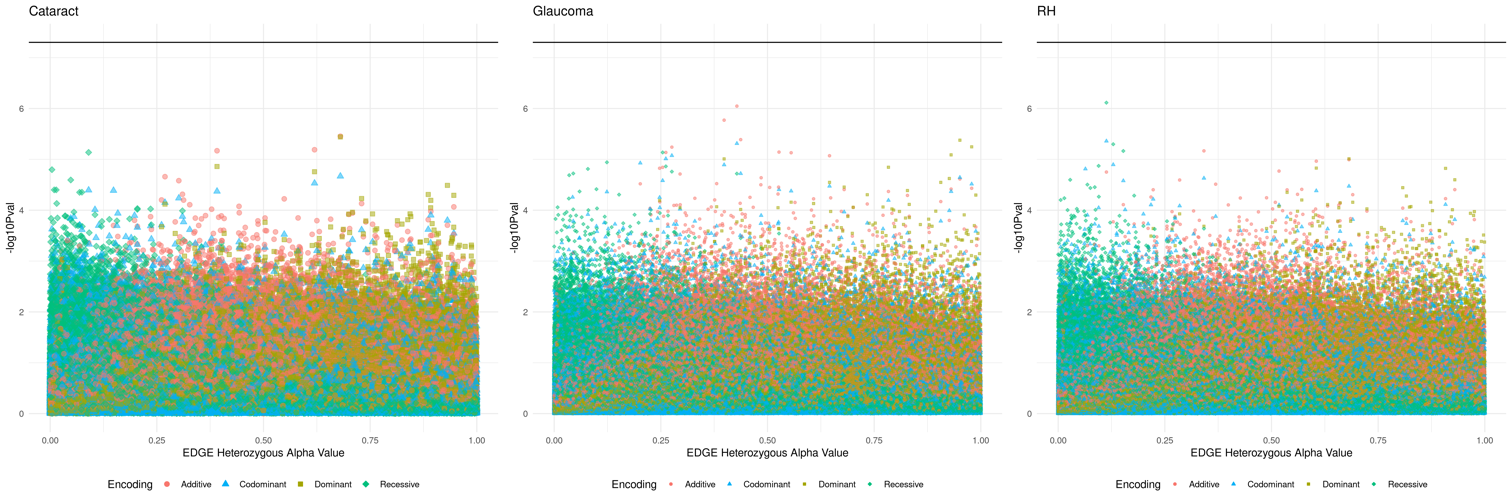

Supplement: S2 Fig — (DOCX) [file pgen.1009534.s003.docx]
