# Supplementary material for: Novel EDGE encoding method enhances ability to identify genetic interactions
Source: PLoS Genet. 2021 Jun 4;17(6):e1009534. doi: 10.1371/journal.pgen.1009534 (PMC8208534; doi:10.1371/journal.pgen.1009534)
Supplement: Attachment — Submitted filename: Response-to-Reviewers.docx [file pgen.1009534.s016.docx]

**Response to Reviewers**

Reviewer #1: Hall et al. present a new "elastic data-driven genetic encoding" (EDGE) method to enhance identification of genetic associations and epistatic interactions. Instead of being restricted by various genetic model parameters of the heterozygous genotype, EDGE concerns a single parameter alpha as the ratio of the linear regression coefficients of the heterozygous and the homozygous alternate genotypes, and thus is elastic and data-driven. Based on a set of simulation and real data analyses, the authors conclude that the EDGE method is robust and can be used as a flexible alternative for testing pair-wise epistatic interactions among common variants.

While the idea is intriguing, the manuscript is not easy to read and the work seems incomplete with a few key questions can be asked. What concerns me most is that none of the identified epistatic interactions are tested for replication in an independent data. I hope the following comments are useful to improve the manuscript.

**We thank the reviewer for their comments. We will respond to the concerns in detail below. Briefly, further clarity and details have been added throughout the manuscript for readability, and we looked for replication of our interaction results in the UK Biobank (UKB).**

Major points:

Replication. Statistical epistatic interactions remain a challenging topic for many reasons. One reason is lack of robust examples with strong statistical support and validated biological meanings. Statistical replication is an important first step to prevent reporting false positive findings. Considering just the rs7787286 – rs4695885 interaction in age-related cataract identified only by EDGE, the MAF of rs7787286 is 0.035 in Europeans that is lower than 0.05 used in the simulation scenarios and the QC procedures of eMERGE data. Statistical replication is therefore recommended.

**Thank you for this. We couldn’t agree more. As such, we looked for replication of our SNP-SNP interaction results. Epistatic signal is challenging to detect, and replications of the exact SNP-SNP pairs are even more difficult as they can be influenced by difference in MAF and LD structure. Other papers have successfully attempted gene-based replication, such as Verma *et al*.; however, interaction SNPs associated with age-related cataract are not located in genes, so we chose to perform a region-based replication, and this has been added to the manuscript. Three of the exact SNP-SNP pairs replicated in UKB (for AMD and resistant hypertension). For cataract, we identified an alternate SNP-SNP pair within the 25kb region of the original SNPs. Unfortunately, the interaction associated with T2D in eMERGE did not replicate in UKB. These details were added to the Results and Methods sections of the manuscript.**

**Verma SS, Cooke Bailey JN, Lucas A, Bradford Y, Linneman JG, Hauser MA, et al. (2016) Epistatic Gene-Based Interaction Analyses for Glaucoma in eMERGE and NEIGHBOR Consortium. PLoS Genet 12(9): e1006186. https://doi.org/10.1371/journal.pgen.1006186**

Low frequent variants. As mentioned in the Discussion section, EDGE currently concerns only frequent variants and the authors recommend caution when working with low frequent variants, i.e. MAF less than 10% if I understand correctly. However, recent theoretical studies suggest that common variants identified in GWAS are often associated with high LD and low probability of being causal. It seems necessary to cover low frequent variants in epistasis studies although this means large samples are required to identify their interactions.

**This is a great point, and in fact, in this study we did consider low frequency variants in the eMERGE data. We often use a 5% MAF filter in our QC pipelines to reduce the number of tests, knowing we often do not have power to detect SNP signal using the additive encoding. However, we thought it possible that EDGE may be able to detect interaction between SNPs with lower MAF than 5% and did not apply the MAF QC filter, even with the higher multiple testing burden that would be required. While this was not the main focus of the paper, given the focus of simulating SNPs with 10%, 30%, and 50% MAF, the SNP-SNP interaction for cataract mentioned in the comment above involved rs7787286, which was low frequency in eMERGE as well (0.041). Given the significant interaction for cataract, we see EDGE as a potential method for exploring low frequency variants for epistasis. Future work will focus on evaluating EDGE with low frequency variant simulations and applying it to low frequency variants in natural data. Additionally, it is important to note that we do not state that any of the signals we find are causative, and we added to the discussion that signals identified could be due to the SNPs being in high LD with functional variant. We see our method as an important approach to explore the wealth of SNP data available from the plethora of GWAS that have already been performed. We believe that adding EDGE to the genetic association toolbox for epistasis will reveal further genetic interactions that deepen our understanding of the complex etiology of disease. All of this information has been revised and discussed in the Methods, Results and Discussion sections.**

Simulation. If I understand correctly, the simulation scenarios used concern only one pair of SNPs simulated with predefined parameters, but without concerning the genome-wide settings incurred in real data analyses. I think the impact of this limitation needs to be discussed as well.

**Thank you very much, and we agree that epistasis is likely to be far more complex than pairwise SNP-SNP interactions. Assessing one pair of SNPs at a time was our first step because consideration of pairwise SNPs is the common approach when studying interactions. Our first step was to evaluate our method compared to conventional encodings in the way they are typically utilized. Future work will involve modeling more complex interactions in the context of the genome, including multiple interacting loci. This limitation has been added to the Discussion.**

**Additionally, one other point that is worth noting is that we did not embed the signals into datasets of genome-wide SNPs with random effects. In these analyses, the tests are exhaustive, meaning that every SNP-SNP pair is evaluated. Since we would test every SNP pair, the importance of the simulation is not the feature selection step – which is where having hundreds of thousands of noise SNPs would be worthy of evaluating. The goal of simulation here is testing the modeling ability of the method. Adding hundreds of thousands of noise SNPs would have increased the computation time without increasing the interpretation of the results. It is important to note that when running genome-wide or on large sets of SNPs, correction for multiple testing is essential.**

Significance threshold. I am a bit nervous about the ways of deriving significance thresholds. I can understand using EDGE as a main effect filter for interaction tests but can understand why using the top 50 (not 30 or 70 for example) most significant SNPs per model to derive the test set, which appears to assume EDGE alone could pick up epistatic SNPs without bias and could lead to varied size of the test set and subsequently jeopardize Bonferroni adjustment.

**We did not use EDGE to main effect filter SNPs. Rather, traditional encodings (additive, dominant, recessive, and codominant) were used for the main effect filter. We discussed the results of these multi-encoding GWAS in the paper but also used them as a way to filter for SNP-SNP interactions. Clarity of this has been added to the Methods and Results. As for the number of SNPs chosen for subsequent interaction (top 50 SNPs from each encoding GWAS), we chose this number so to allow a relatively large number of interacting SNPs while maintaining a manageable multiple test and computational burden. We used the same number of SNPs for each encoding, as opposed to a significance threshold, so as not to bias any one encoding type if one encoding yielded more results from the GWAS. Greater clarity for this has also been added to the methods.**

Minor points:
• For each pair of epistatic interacting SNPs, please report MAF of each and their LD given the pruning QC at 0.7
• Please add detailed MAF information in the Abstract
• The Introduction could be stronger if updates of recent development in studying epistasis could be provided. I am a bit surprised that only 14 references are cited for this difficult topic
• page 3 line 64: 'appled' to 'applied'
• page 5 lines 106 to 107: what are 'the current state-of-the-art methodologies'? what is the current paradigm?
• page 5 lines 108 to 110: 'Results discussed ... for GxG' – move the statement to Discussion?
• page 6 Table 2: 'Codominant (HA)' to 'Homozygous alternate (HA)'
• page 8 line 163: the dominant scenario includes under- and over-dominance?
• page 8 lines 169 to 172: in Figure 1 why super-additive varied a lot more than sub-additive and additive? why presenting only MAF of 0.3?
• page 9 lines 190 to 197: in Figure 2 the genotype model seems doing ok at the LRT cutoff of 0.05?
• line 221: ';,' to ';'
• line 232 and line 242: '50% (B)' to '50% (C)'?
• lines 246 to 262: what MAF in the NULL simulations?
• lines 272: please provide scales for the signal to noise ratios in Figure 7
• line 294: 'demonstrated similar patterns' – sorry but I can't tell
• line 304: 'uncorrected p-value' – why not using corrected p-value instead in Figures 8 to 10
• line 352: rs117537110 not available in dbSNP, a typo?
• lines 515 to 516: 'it is not expected ... under this scenario' – not sure about this claim, perhaps remove it?
• line 613: please specify if LRT p-values are derived using the genotype encoding or other settings, with consistent degrees of freedom.

**Thank you very much for all of these points. Each have been addressed and updated in the manuscript.**

**Regarding the question about rs117537110: We do find this SNP in dbSNP in PPP1R18. We have double checked our results and genotype files. This does not appear to be a typo.**

Reviewer #2: Thank you for the opportunity to review the work. EDGE is a robust method to test for SNP-SNP interaction regardless of underlying encoding for SNPs. The method has use beyond this application and can also assess what the true underlying encoding a SNP has. The authors present thorough simulations and a very nice application to the eMERGE dataset. Interestingly, they show that in certain situations, SNPs with additive encoding can have inflated type I error for interaction. This is the most common encoding used in the field and EDGE is robust to this. While the simulations and results are strong, the presentation of the results can be improved, especially for readers that have difficulty with discerning colors from one another.

**Thank you for providing us with insightful comments. Below we will respond to each comment in further detail.**

1. Type I error is usually presented before power estimation and is helpful to show that the methods maintain appropriate type I error. I suggest moving this section to the beginning of Results.

**Thank you, we have moved this section above the simulated interaction results in the manuscript.**

2. Figure 3/Line 615. It is unclear to me how the authors assess the “significance of parameters” such as MAF and what the LRT is testing. More detail is needed for this.

**We apologize for the confusion. In the Methods, the description of LRT was regarding the interaction tests and not the parameter sweep. We have added sub-headings to these paragraphs so as to ensure this is clearer. To test the impact of allele frequency, sample size, penetrance, and case-control ratio on power, we performed a parameter sweep. All 29 interacting models were simulated with comprehensive combinations of each of the four parameters. After compiling all power results from simulated datasets of our parameter sweep, we performed ANOVA tests of the power by model for each encoding to identify effects of each parameter on power for every genetic model type. We have also added more clarification to this section you noted as well as in the Methods. We hope that this added clarity to these components.**

3. Figures 4 and 5. Different types of dashed lines would be useful to supplement the different colors for the lines.

**This has been updated for these figures.**

4. Why does codominant model show reduced power with highest signal to noise? The other methods show much more what I would expect, increased power as signal grows.

**Yes, this is something we continue to see with the codominant encoding. As the signal increase (and/or MAF decreases), the power decreases due to model non-convergence, which the codominant (dummy) encoding is particularly prone to. We have added this to the description of these figures in the manuscript.**

5. Figure 6. Add dotted vertical lines for Bradley’s liberal criteria to better show which points violate this and thus don’t have correct type I error.

**This is a helpful suggestion. We have added these lines.**

6. Line 271. Clarification is needed for Figure 7 to make sure the reader knows that the max/average is being assessed for all of the different scenarios assessed in Figure 6.

**Thank you, we agree that clarification was needed and we have added more details to the description of Figure 6.**

7. Figure 7. Dashed black line gets confused with other dashed lines and the legend. Please change to dotted and make sure to add to legend.

**We agree with the reviewer and as we inspected this further believe that the upper black line was too arbitrary and chose to remove it altogether.**

8. Figures 8-10. Please use same symbols as in Figure 1 to further distinguish the encodings.

**Thank you for this excellent suggestion. This has been updated.**

9. Figure 10 can be moved to Supplement and Figures 8 and 9 can be combined in one plot. Fig 8 and 9 get the point across well and nicely visualize alpha estimated from EDGE.

**This has been updated.**

10. Suggestion to add two figures (T2D and AMD) which make pairwise comparisons of the –log10p for different encodings shown in Figures 8-10. See below made up example.

-log10p for REC

-log10p for ADD

**We have added this plot to the paper as well as a description. Thank you for suggesting this excellent addition to the paper.**

11. Line 349. Should be SNP-SNP interaction and not gene-gene

**This has been changed.**

12. Figure 11-14. Better labels for tracks are needed: SNP1 alpha, SNP2 alpha, -log10p interaction

**We have updated this.**

13. Why are there outlier alphas in Figures 12 and 13? What is the interpretation here?

**We developed EDGE to have the flexibility to assign a heterozygous alpha value to SNPs that is outside of the 0-1 range in the scenario that SNP may act in 1) an over-dominant manner (alpha > 1) where the heterozygous risk exceeds the homozygous alternate risk or 2) an under-dominant manner (alpha < 0) where the heterozygous risk is less than the risk of being homozygous for the referent allele. As we see in these figures, there is evidence of SNPs acting outside of the 0-1 range. We see this as further importance of adding EDGE to the genetic association toolbox because of its flexibility. We have added further clarity to this when discussing these figures in the manuscript and when introducing the possible SNP genetic models.**

14. Figure 13 has labels but the other similar plots don’t. I suggest removing these to make the figures uniform across different traits considered.

**This has been updated.**

15. Using the union of the top 50 SNPs is very arbitrary and deserves discussion. Others have suggested using p-value thresholds to filter out SNPs with main effect for testing interaction.

**Thank you for pointing this out. We chose this number so to allow a relatively large number of interacting SNPs while maintaining a manageable multiple test and computational burden. We used the same number of SNPs for each encoding, as opposed to a significance threshold, so as not to bias any one encoding type if one encoding yielded more results from the GWAS. Greater clarity for this has also been added to the methods**

16. GxE interaction has become very popular in recent years. It would be useful for the authors to expand a sentence or two in the discussion on how EDGE could be useful in this context and how it would need to change (if it needs to be modified).

**We are thrilled to hear you see the benefit of EDGE in GxE interaction research, as this is an area of focus for Dr. Hall as she is growing her research program. Applications of EDGE to GxE are in development, and further discussion has been added including key areas that need to be explored in the context of the exposome, including: 1) how the alpha value may vary across different environmental exposure contexts and 2) the impact of exposure variable type (i.e., categorical versus continuous).**

Reviewer #3: This paper proposes a new and more flexible encoding method named EDGE to assign coefficients to the heterozygous alleles for different SNPs. The authors claim that this new encoding method provides higher power in detecting SNP-SNP interactions than existing methods which make rigid assumptions about the underlying interaction models. Experiments using simulation and real data are shown to illustrate the claim.

In GWAS, there are at least about 51 different SNP-SNP interaction models [Li and Reich 2000]. Enumerating all these models is indeed a difficult task. The motivation of this paper is very nice. There are, however, some issues that the authors may wish to address:

1. It is unclear to me how the authors infer the right model for each interacting SNP-SNP pair. If they just use Eq. (1) and Eq. (2), then probably the authors will decide the model type based on the value of \alpha. In table 2, the authors have shown some typical examples of additive model, dominant model, recessive model, etc. I notice that the \alpha values in these models are separated at least by 0.25. It is unclear if the authors will choose the model type based on this interval or based on the actual \alpha value. Also, it is unclear if the authors will carry out any model validation.

**Thank you for this comment. We have added clarity to the description of Table 2. The purpose of Table 2 is to illustrate the assumptions that the traditional methods make (e.g., additive assumes having one copy of the minor allele yields precisely half the risk of having 2; Dominant assumes one and two copies of the minor allele yield the same risk) and to compare that with what we believe is the advantage of EDGE: that EDGE assigns a flexible heterozygous encoding (alpha). We can see that the original manuscript was not clear about this and we have added clarification throughout the Introduction and Results. As for validation, we looked for replication in the UK Biobank for all of the SNP-SNP interaction models and found a number did replicate.**

2. The power analysis and the comparison with existing methods need to have more elaboration. For example, how much of the power gain is due to the flexibility of EDGE in model determination (based on \alpha value estimation) and how much is due to the detection method itself? This could be demonstrated in the simulation experiments by generating simulation data with a well-defined interaction model and then compare the performance of EDGE and that of existing methods which assume exactly the same interaction model as in the simulation.

**Thank you. This description of simulation testing is what we did for our simulation analysis. We have added more details and clarification throughout the Methods and Results sections of the paper to ensure this is clear to readers.**

3. There are already some widely-used SNP-SNP interaction detection methods, such as PLINK, MDR, BEAM, and BOOST. The authors should show comparison results with respect to these methods to showcase the advantages of their new method EDGE.

**Thank you very much for pointing this out. We agree that there are multiple alternative SNP-SNP interaction methods beyond regression (e.g., MDR, BEAM, ATHENA). Because the goal of this paper is to provide a novel and flexible encoding method for regression-based SNP-SNP interactions, we see the first step as to compare EDGE to existing encodings (additive, dominant, recessive, and codominant encodings) within the regression framework. Future work will involve extending EDGE to machine learning interaction methodologies which will involve rigorous assessment and comparison to these methods. A study comparing of MDR to EDGE is already underway. Regarding BOOST, the method encodes SNPs in a codominant manner, but its methodology is unique from the approach we used to assess the encodings in this paper. In a separate project, authors Dr. Hall, Dr. Van Steen, Dr. Ritchie, and Dr. Müller-Myhsok are performing an in-depth comparison of EDGE and BOOST, and this paper is in preparation. A discussion of this has been added to future work portion of the Discussion section of the paper.**

**Finally, PLINK interactions are performed using additive, dominant, recessive, or codominant encodings (by user preference) and is performed in the same way as our PLATO software, which was used in the current study. The only difference is that PLATO has the additional optional EDGE encoding.**

Reference:
Li W, Reich J: A complete enumeration and classification of two-locus diseasemodels. Hum Hered 2000, 50:334–349.
